# Log-ratio transformations for dietary compositions: numerical and conceptual questions

**DOI:** 10.1017/jns.2021.93

**Published:** 2021-11-15

**Authors:** Maria Léa Corrêa Leite

**Affiliations:** National Research Council/Institute of Biomedical Technologies, Milan, Italy

**Keywords:** Compositional data, Dietary data, Energy intake, Log-ratio transformation, Macronutrients, Nutrient balances

## Abstract

When evaluating the impact of macronutrient intakes on health outcomes, researchers in nutritional epidemiology are mostly interested in two types of information: the relative importance of the individual macronutrients and the absolute effect of total energy intake. However, the usual substitution models do not allow these separate effects to be disentangled. Dietary data are typical examples of compositional data, which convey relative information and are, therefore, meaningfully expressed in the form of ratios. Various formulations of log-ratios have been proposed as a means of analysing compositional data, and their interrelationships when they are used as predictors in regression models have been previously reported. This note describes the application of distinct log-ratio transformations to the composition of dietary macronutrients and discusses the interpretative implications of using them as explanatory variables in regression models together with a term for the total composition (total energy intake). It also provides examples that consider serum glucose levels as the health outcome and are based on data coming from an Italian population-based study. The log-ratio transformation of dietary data has both numerical and conceptual advantages, and overcomes the drawbacks of traditional substitution models.

## Introduction

Isocaloric substitution analysis has been considered the gold standard for nutritional studies aimed at evaluating the relationships between macronutrient intake and the risk of disease because it provides a means of assessing the effects of replacing specified nutrients on a health outcome while adjusting for total energy intake.

For example, taking *y* as a health outcome, EC, EP and EF as respectively representing the dietary intakes of energy coming from carbohydrates, proteins and fats, and TE as their sum (total energy intake), the isocaloric substitution model that leaves out EF would be:
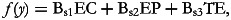
in which B_s1_ is interpreted as expressing the specific effect of replacing fats with carbohydrates while keeping total energy and protein intakes constant. Alternatively, the energy partition model:
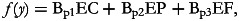
can be used to obtain replacement effects by estimating them as the difference between B_p_ coefficients. These approaches are equivalent, as it can be easily seen that B_s1_EC = B_p1_EC – B_p3_EF, B_s2_EP = B_p2_EP – B_p3_EF and B_s3_TE = B_p3_EF.

Arnold *et al.*^(1)^ have recently provided a conceptual description of two potential types of causal effects for compositional data such as dietary data. In their case, B_p(s)_ represents the *total effects* (not conditioned on TE) of adding each macronutrient to the diet regardless of the intake of all of the other macronutrients, consequently increasing total energy ‘without altering other consumption behaviours’, while B_s(s)_ represents the *relative effects* arising from the isocaloric replacement of fat with carbohydrate (B_s1_) or protein calories (B_s2_). These are, therefore, the joint effects of reducing fat consumption and increasing the consumption of other macronutrients. In brief, the authors assume that the interdependence of macronutrient effects arises from the conditioning on total energy intake.

However, these models cannot unequivocally disentangle the different effects of macronutrients and total energy intake because they are unsuitable for describing the intrinsic nature of variations in dietary components. Although they overcome the numerical problem of perfect data collinearity, they raise a conceptual issue: the compositional nature of the data means that the information conveyed by the compositional parts is inherently relative regardless of the inclusion of their total in the model, and so any attempt to estimate the effects of isolated variations can lead to misleading results.

In order to illustrate some concepts intuitively and schematically, let us consider a typical daily diet of 2000 calories, of which 55 % come from carbohydrates 15 % from proteins and 30 % from fats (diet A in Table 1). The macronutrient composition represents the qualitative aspect of the diets and the total calories (last column) its quantitative aspect.

**Table 1.** Characteristics of four fictitious diets

Diet	Macronutrient composition: kcal (%)			Total calories (%)
	EC	EP	EF	TE
A	1100 (55·0)	300 (15·0)	600 (30·0)	2000 (100·0)
B	1400 (61·0)	300 (13·0)	600 (26·0)	*2300 (100·0)*
C	1400 (70·0)	300 (15·0)	*300* (*15·0)*	2000 (100·0)
D	1100 (48·0)	300 (13·0)	*900* (*39·0)*	2300 (100·0)

The only difference between diet B and diet A is the increase in the number of calories coming from carbohydrates. In the framework of an energy partition model, interpreting the effect of adding EC as the only variation is somewhat misleading because the consequent increase in total calories (in italics to indicate its exclusion from the model) leads to changes in the ‘weights’ of the components of the composition: for example, the 26 % of calories coming from fat in diet B is qualitatively different from the 30 % in diet A. In diet C, calories from carbohydrates isocalorically replace those coming from fats in diet A, the effect of which would be estimated in a substitution model by including the term for total energy intake and excluding that of EF. However, this is not the only qualitative change because the relationship between protein and fat content substantially changes from 1:2 in diet A to 1:1 in diet C, thus raising the question of whether it is metabolically irrelevant. Finally, diet D illustrates how the variation in TE coincides entirely with the variation in the component left out of the model: this is actually the only component free to vary, and therefore, the only component contributing to the increases in total energy intake. It is, thus, clear that substitution methods cannot provide appropriate estimates of the effect of total energy intake.

Nutrition researchers may not only be interested in obtaining isocaloric estimates of nutrient effects but also in achieving a meaningful estimate of the effect of total energy intake regardless of the qualitative nature of a diet (particularly, in the context of obesity and related metabolic disorders). In order to illustrate this intuitively, let us consider three diets (E, A and F in Table 2) in which the absolute (quantitative) aspect increases and these changes are fully represented by the total number of calories, while their qualitative aspect (macronutrient composition) remains unchanged. Although these diets are qualitatively similar, it can be seen that the differences between the amounts of macronutrient increase together with the total number of calories, for example, the difference between the calories coming from fat and the calories coming from proteins is 150 in diet E, 300 in diet A and 450 in diet F. It is, therefore, clearly impossible to disentangle the quantitative and qualitative aspects of the diets on the basis of the differences between their compositional parts. On the other hand, the relationships (ratios) between the parts remain constant in all three diets, and it would, therefore, seem to be logical that ratios are more suitable than differences when it comes to describing the characteristics of a composition.

**Table 2.** Characteristics of three fictitious diets

Diet	Macronutrient composition: kcal (%)			Total calories (%)
	EC	EP	EF	TE
E	550 (55·0)	150 (15·0)	300 (30·0)	1000 (100·0)
A	1100 (55·0)	300 (15·0)	600 (30·0)	2000 (100·0)
F	1650 (55·0)	450 (15·0)	900 (30·0)	3000 (100·0)

The basic principle of compositional data analysis is that ‘any meaningful function of a composition can be expressed in terms of ratios of the components of the composition’^(2)^ insofar as ratios are the natural means of describing variations that are intrinsically relative.

## Log-ratio transformations of compositional data

Compositional data can be defined as positive vectors of parts of a whole that convey relative information^(3)^. Given that interest lies in the relative amounts of compositional components and that logging the ratios is a convenient means of making them more easily manageable mathematically, various expressions of log-ratios have been proposed for the analysis of compositional data.

Log-ratio transformations produce new variables that are amenable to being analysed using standard statistical methods, and a family of transformations has been introduced within the framework of log-ratio methodology. In the case of a D-part composition (*x*_1_, *x*_2_, … , *x_D_*), the additive log-ratio (alr) transformation involves the division of each *D*−1 component by one that is arbitrarily chosen, for example, the last:
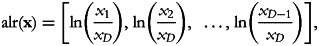
while the centred log-ratio (clr) transformation is defined by
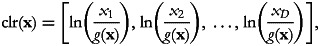
where *g*(**x**) is the geometric mean of the components of the composition^(4)^.

Subsequently, Egozcue *et al.*^(5)^ proposed the isometric log-ratio (ilr) transformation which, in its particular expression of *balances*, consists of orthogonally decomposing the parts of a whole into non-overlapping subgroups and representing their relationships. One way of constructing orthonormal balances is to use sequential binary partition (SBP) as a bifurcating tree: the parts of a composition are successively and hierarchically split into two groups until all of the groups have a single part^(6)^. At each of the *D*−1 steps required to complete the partition, a generic balance is defined as the orthonormal log-ratio of the geometric mean of each group of parts:
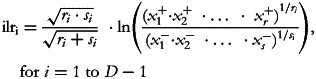
where the square root coefficient is the normalising constant, and *r* and *s* are respectively the number of parts in the numerator (*x*^+^) and the number of parts in the denominator (*x*^−^).

One particular choice of partition has been proposed^(7)^ in which the ilr transformation is defined as:
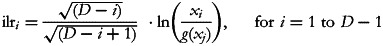
where *g*(*x*_*j*_) is the geometric mean of the compositional parts for *j* = *i* + 1 to *D*. The particular characteristic of this approach is that the first new variable (ilr_1_) captures all of the relevant information about the compositional part *x*_1_, and is, thus, called the *pivot balance*^(8)^. A number of *D* sets of *D*−1 ilr(s) are defined, each of which has a different compositional part in the numerator of the pivot balance.

## Macronutrient log-ratios as explanatory variables

This section describes how these different log-ratio transformations can be applied to dietary macronutrient compositions and examines the interpretative implications of their use as explanatory variables in regression models, on the basis of some elements that have recently been discussed^(9)^. To this end, imagine a five-part composition of dietary proteins (PR), starches (ST), simple sugars (SS), unsaturated fats (UF) and saturated fats (SF) as sources of energy, and then consider the definition of balances we have previously suggested as a means of characterising dietary exposure^(10–12)^. The partitioning procedure can be carried out using the following sign matrix, where the plus sign indicates that the part is assigned to the numerator of the balance, the minus sign indicates that it is assigned to the denominator, and 0 indicates that it is not involved in that particular balance:

**Table:** 

PR	ST	SS	UF	SF
−1	−1	−1	+1	+1
+1	−1	−1	0	0
0	+1	−1	0	0
0	0	0	+1	−1

The corresponding balances can be represented as explanatory variables as follows:
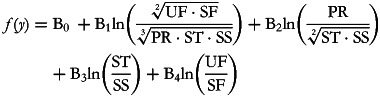
but note that the normalising constant has been removed, consequently reducing the orthonormality of the log-contrasts to simple orthogonality. This simplification has been previously proposed by Muller *et al.*^(13)^, in order to enhance the interpretability of the results of regression analyses involving ilr coordinates as explanatory variables.

As in any multiple regression framework, one B-coefficient represents the change in response associated with a one unit change in the corresponding log-ratio, while keeping all of the other log-ratios constant. Adding on the log scale is equivalent to multiplying on the natural scale and since 1 = ln(*e*), B_1_ therefore represents the expected change in the dependent variable when the ratio between the geometric mean of the dietary fats and the geometric mean of the other macronutrients (proteins and carbohydrates) is multiplied by *e* (approximately 2·7). It is worth noting that using the binary logarithm (base 2) may often be useful as it corresponds to the effect of doubling the ratio. This effect may be subject to intrinsic confounding because an increase in fat consumption may variously involve saturated and/or unsaturated fats and, similarly, a decrease in the denominator may be variously related to proteins and/or carbohydrates. However, these confounding factors can be appropriately controlled by including in the model the other balances, whose embedded ratios are kept constant. This ensures that variations in the B_1_-related ratio are made in such a way as to increase UF and SF by a common factor and decrease PR, ST and SS by a common factor. As another example, B_4_ expresses the effect of multiplying the UF:SF ratio by 2·7 without modifying the relationships between the other macronutrients as represented by the ratios in the equation.

Using the simplified (orthogonal rather than orthonormal) pivot balance approach, five regression equations can be drawn up by defining the pivot balance for each of the five components and using five runs. The first is:
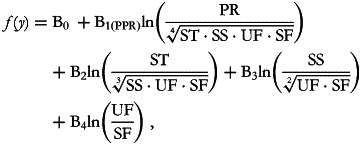
the second is:
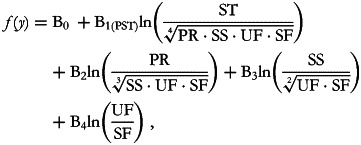
and so on. At each run, we are interested in inferences about the first balance which captures all of the available information about the part in its numerator. Thus, B_1__(PPR)_ represents the expected change in the dependent variable when the balance between proteins against all of the other macronutrients is multiplied by an *e*-factor of 2·7. This variation is the result of increasing the protein component while reducing all of the other parts by a common factor as is ensured by the model's inclusion of the other balances. The B_1(PST)_ coefficient is interpreted in the same way but concerns starches.

Let us now consider the model that includes four alr(s) as predictors:
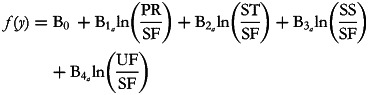
The coefficient 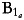 represents the expected change in outcome when PR increases and SF decreases in such a way that the PR:SF ratio increases 2·7 times, while keeping all of the other terms in the model constant. This necessarily means that ST, SS and UF decrease by the same factor as SF. Thus, although the other parts are not explicitly present in the denominator of the ratio, 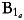 measures the same effect as B_1__(PPR)_ and 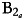 measures the same effect as B_1(PST)_ and so on.

Finally, consider the case of a model including clr(s) as explanatory variables. Because of the perfect collinearity 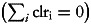, one clr (for example, the one with SF in the numerator) is left out of the equation:
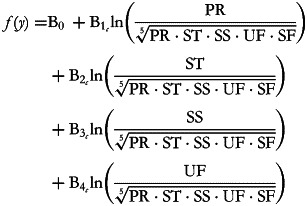
Increasing a given clr while keeping the remaining log-ratios in the equation constant requires a decrease in the omitted clr by the same amount: for example, adding one unit [ln(*e*)] to ln(PR/gm), where gm is the geometric mean of the five components, implies reducing ln(SF/gm) by the same amount, and as a result, the difference [ln(PR/gm) – ln (SF/gm)] = ln(PR/SF) increases by 2 ln(*e*). That is to say, 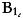 represents the expected change in the dependent variable when the ratio between PR and SF is multiplied by 2·7^2^, and this also applies to the other regression coefficients in the equation. Curiously, in this case, although the denominator of the ratios includes all of the compositional parts, the estimated effects are related to pair-wise ratios.

## Accounting for total energy intake

As said in the introduction, when investigating the relationship between dietary macronutrient composition and health outcome, nutritional researchers may be interested not only in examining the isocaloric effects of relative variations in macronutrient intakes, but also in obtaining a meaningful estimate of the effect of total energy intake. In other words, not only the relative size of the compositional parts but also their absolute variable size may be relevant.

The statistical properties of alternative ways of computing a total of compositional observations have been described by Pawlowsky-Glahn *et al.*^(14)^, who have stated that a product-based total such as 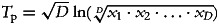 is isometric (which means that it preserves the same distances as in the logarithm value space), but a sum-based total such as *T*_s_ = ln(*x*_1_ + *x*_2_ + · · · + *x*_*D*_), which looks more like what is usually understood as a total, can also be used.

Since then, Coenders *et al.*^(15)^ have discussed cases in which a composition and its total act as explanatory variables and described the interpretative implications of using these different formulations of the total. Going back to our five-part macronutrient composition, the alternative models containing four balances and a total are:
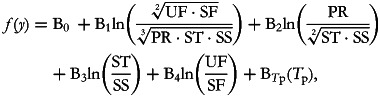
where 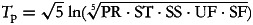, and

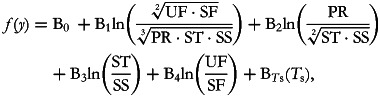
where *T*_*s*_ = ln(PR + ST + SS + UF + SF) = ln(total energy)

Both B*_T_*_p_ and B*_T_*_s_ express the effect of increasing the overall size of the macronutrient composition while keeping the relative importance of its component parts constant: i.e., they increase in the same proportion. However, while B*_T_*_p_ is related to multiplicative changes in the not very intuitive quantity given by (PR·ST·SS·UF·SF)^1/√5^, B*_T_*_s_ is more simply related to multiplicative changes in total energy intake. The interpretation of the balance-related coefficients B_1_ to B_4_ changes only slightly depending on which *total* is held constant. As the coefficients refer to the effect of increasing the relative balance while keeping the remaining terms constant, it implies that all of the parts in the numerator of the balance increase in the same proportion and those in the denominator decrease in the same proportion. Furthermore, if the total included in the model is B*_T_*_p_, the increase in the numerator of the balance is exactly counteracted by the decrease in the denominator. This perfect offset is not assured when the sum-based total (B*_T_*_s_) is used, but the fact that B*_T_*_p_ can be mathematically considered the ‘true total’ does not mean that using B*_T_*_s_ is a worse choice. In the particular case of dietary data, total energy intake as a sum of the calories from the different macronutrient sources is a significant characteristic of study subjects. Moreover, isometry is not a requirement when using compositions as explanatory variables.

## Example: dietary macronutrients and serum glucose levels in non-diabetic subjects

This example uses data from the Italian Bollate Eye Study^(16)^, a population-based study involving participants aged 40–74 years that was carried out in 1992–1993. The participants’ dietary habits were assessed by means of a food-frequency questionnaire, and their mean daily nutrient intakes were calculated using the food compositional database compiled for epidemiological studies in Italy^(17)^. The study was approved by the Ethics Committee of the Italian National Research Council (CNR).

Various linear regression models with serum glucose level as the dependent variable were fitted and, as the concentration units (mg/ml) indicate compositional information, their values were logged in order to allow an estimate of relative changes. In addition to the dietary variables (total energy and fibre intake, and macronutrient log-ratios), all of the models included terms for gender, age, practising sport, television watching time, smoking and alcohol consumption. In order to simplify the interpretation of effect size, logarithms of the independent dietary variables were computed using base 2 [remember that log_2_(*z*) = ln(*z*)/ln(2)], and so the coefficients represent the effect of doubling the amounts of calories, fibre and the ratio in question. Tables 3–6 show the results of running the models, including a different formulation of the log-ratios or the total each time. The examination of the residual plots did not show any particular trend in their distribution and did not reveal deviations from linearity or homocedasticity, thus indicating that the models are adequately specified (data not show). As has been previously shown^(9)^, the different formulations of the log-ratios represent simple reparametrisations of the same model and, as a result, the models performed equally and the estimates related to covariates common to the different models were the same.

**Table 3.** Results of the linear regression analysis of serum glucose levels [ln(mg/ml)] in relation to total energy and fibre intake and macronutrient balances (orthogonal coordinates)

	Coefficient	Standard error	*P*-value
Model A			
Total energy (*T*_p_)	−0·0038	0·0078	0·627
Total fibre	0·0191	0·0144	0·186
Macronutrient balances:			
Fats *v.* proteins, carbohydrates	0·0195	0·0131	0·135
Proteins *v.* carbohydrates	0·0433	0·0130	0·001
Starches *v.* simple sugars	−0·0069	0·0061	0·256
Unsaturated *v.* saturated fats	−0·0105	0·0136	0·442
Model B			
Total energy (*T*_s_)	−0·0030	0·0173	0·860
Total fibre	0·0154	0·0145	0·288
Macronutrient balances:			
Fats *v.* proteins, carbohydrates	0·0169	0·0120	0·158
Proteins *v.* carbohydrates	0·0435	0·0131	0·001
Starches *v.* simple sugars	−0·0075	0·0063	0·236
Unsaturated *v.* saturated fats	−0·0083	0·0133	0·533

**Table 4.** Results of the linear regression analysis of serum glucose levels [ln(mg/ml)] in relation to total energy and fibre intake and macronutrient balances (simplified pivot coordinates)

	Coefficient	Standard error	*P*-value
Total energy (*T*_s_)	−0·0030	0·0173	0·860
Total fibre	0·0154	0·0145	0·288
Pivot balance (the first coordinate in each of the five runs)^a^:			
Proteins *v.* other macronutrients	0·0378	0·0155	0·015
Starches *v.* other macronutrients	−0·0349	0·0090	0·000
Simple sugars *v.* other macronutrients	−0·0199	0·0080	0·014
Unsaturated fats *v.* other macrontrients	0·0002	0·0134	0·990
Saturated fats *v.* other macronutrients	0·0167	0·0156	0·284

a Each run involves four balances, of which the first is the pivot.

**Table 5.** Results of the linear regression analysis of serum glucose levels [ln(mg/ml)] in relation to total energy and fibre intake and macronutrient additive log-ratio (alr) coordinates

	Coefficient	Standard error	*P*-value
Total energy (*T*_s_)	−0·0030	0·0173	0·860
Total fibre	0·0154	0·0145	0·288
Alr-coordinates:			
Proteins *v.* saturated fats	0·0378	0·0155	0·015
Starches *v.* saturated fats	−0·0349	0·0090	0·000
Simple sugars *v.* saturated fats	−0·0199	0·0080	0·014
Unsaturated fats *v.* saturated fats	0·0002	0·0134	0·990
Saturated fats *v.* unsaturated fats^a^	0·0167	0·0156	0·284

a Obtained in a different run that included in the model the four alr(s) with unsaturated fats as the denominator.

**Table 6.** Results of the linear regression analysis of serum glucose levels [ln(mg/ml)] in relation to total energy and fibre intake and macronutrient centred log-ratio (clr) coordinates

	Coefficient	Standard error	*P*-value
Total energy (*T*_p_)	−0·0038	0·0078	0·627
Total fibre	0·0191	0·0144	0·186
Clr-coordinates:			
Proteins *v. g*(.)^a^	0·0166	0·0274	0·546
Starches *v. g*(.)	−0·0553	0·0175	0·002
Simple sugars *v. g*(.)	−0·0415	0·0208	0·047
Unsaturated fats *v. g*(.)	−0·0209	0·0272	0·442
Saturated fats *v. g*(.) (omitted)			

a *g*(.): geometric mean of all of the components of the macronutrient composition.

Table 6 shows the results of the regression analysis including the clr(s) coordinates. In this case, the effects should be interpreted as relating to variations in the ratios of each macronutrient against saturated fats, which is in the numerator of the omitted clr. The coefficient for ‘starches *v. g*(.)’ should be interpreted as the effect related to a four-fold (2^2^) increase in the starches:saturated fats ratio. As would be expected, the coefficient relating to a four-fold increase in the unsaturated:saturated fats ratio (−0·0209) is double that of the same ratio estimated in the ilr model (Table 3, model A). Note that the estimates related to total energy (*T*_p_) in Table 6 are the same as those in Table 3.

Table 3 shows the results for macronutrient balances constructed as described above. It can be seen that the inclusion of the different formulations of the compositional total (*T*_p_ in model A and *T*_s_ in model B) had little effect on the fibre- and the balance-related coefficients. A two-fold increase in the protein:carbohydrate balance (shown in such a way that proteins increase and the two types of carbohydrate decrease by a common factor, while all of the other terms remain constant) is related to a expected multiplicative change in the outcome of *e*(0·0435) = 1·0445, where 0·0435 is the regression coefficient estimated for the protein:carbohydrate balance in model B). This means that the serum glucose level is expected to increase by 4·45 % (95 % confidence interval 1·79, 7·17 %).

Tables 4 and 5 show exactly the same results as the differences between them mainly regard the amount of work required. Table 4 shows the coefficients of only the first balance (pivot) that emerged from the five runs, at each of which a set of four balances (corresponding to a distinct partition) was included in the regression equation. Table 5 shows the results of the analysis of the alr transformations. We first included the four alr(s) calculated by dividing each macronutrient by saturated fats and then, in order to obtain estimates for this last, the second run included the four alr(s) with unsaturated fats as the denominator. The ‘starches *v.* other macronutrients’ coefficient in Table 4 indicates that increasing starches against a reduction in the other components by a common factor in such a way that the ratio is doubled leads to a decrease of 3·43 % in the serum glucose level. The identical effect in Table 5 seems to be attributable to the pair-wise ratio ‘starches *v.* saturated fats’, but it is important to note that the inclusion of the other alr-coordinates held constant in the model implies that the other components should decrease in the same proportion as saturated fats; and this also applies to the other alr(s). As would be expected, the estimates related to total energy (*T*_s_) and fibre intake are the same as those shown in Table 3.

## Discussion

On the basis of the modern definition of compositional data, whenever researchers address the relative importance of data components, they are dealing with a compositional problem^(^^3^^)^. The compositional nature of dietary data does not arise from the constraint strictly imposed by conditioning on totals, but is inherent to the dynamics of our way of eating. We do not eat the component parts separately but our food consists of nutrient compositions, and diets rich in some components tend to be rich or poor in others depending on the manner we combine the foods we eat.

Although the compositional nature of dietary data is widely acknowledged, the proposed approaches to compositional data analysis continue to be largely ignored by nutritional analysts. By their very nature, compositional data convey relative information and their expression as ratios is the basic principle of compositional data analysis. Working with ratios is the key difference between compositional data analysis and standard isocaloric substitution modelling which, as it is based on differences and does not allow the relative and absolute aspects of the data to be disentangled, cannot provide a meaningful estimate of the effect of total energy intake.

In this note, we describe the use of different log-ratio transformations of dietary macronutrient composition and discuss the interpretative implications of using them as explanatory variables. As models including different log-ratio formulations provide comparable goodness-of-fit parameters, the choice of which transformation to use should be based on the subject of interest and the interpretative elements. We have previously shown the usefulness of using ilr coordinates in their particular expression as balances as a means of characterising dietary exposure^(12)^. This procedure has the great advantage of being flexibly adaptable to different research questions of interest. Orthogonal balances are new variables that convey non-redundant information and, in the particular case of macronutrient compositions, balances can be easily defined on the basis of sequential binary partitions that follow the components’ natural clustering.

For reasons of simplicity, we have only considered macronutrient compositions but, of course, the questions raised also apply to compositions of micronutrients (vitamins and minerals): for example, researchers may be interested in evaluating the impact of the relative dominance of one vitamin over all of the others. However, although a pivot balance approach may be appropriate, it has the inconvenience of requiring a number of runs that is equal to the number of components in the composition. Nevertheless, as recently pointed out by Coenders^(9)^, alr and simplified pivot coordinates are explanatory equivalents, and as shown in the example, the use of alr transformations as predictors and only two runs lead to the same result as the pivot approach, and so the larger the size of the composition, the greater the amount of work that is saved.

Using dietary log-ratios as explanatory variables not only makes it possible to obtain a meaningful description of the isocaloric interdependence of the components of a dietary composition, but also and simultaneously provides an interpretable estimate of the effect of total energy intake. It is, therefore, possible to evaluate the relative importance of the different parts of the composition while avoiding the fallacious interpretations that may emerge from the raw component analysis. As suggested in the introduction, some attempts to estimate absolute and unconfounded effects may be illusory because changing the concentration of one of the components clearly alters the relationships of the entire composition, while working with ratios provides a means of governing proportional relationships and allows confounding to be finely controlled.

The control provided by the inclusion in the model of the complete set of log-ratios is a key point when examining the explanatory role of a composition. The interpretation of effects may not directly correspond to the way in which the log-ratios are constructed: although their formulation may suggest increasing one component in relation to another (alr) or all of the other components (clr), when they are included as predictors in a regression equation, the related coefficients express the effects of pair-wise ratios (clr) or those in relative terms to all of the components (alr).

In addition to numerical factors, the interest in estimates based on relative variations in dietary elements may also be due to conceptual considerations. In this regard, Kelly *et al.*^(18)^ have suggested a convincing rationale concerning the suitability of using nutrient ratios in nutritional research on the basis of the physiological and metabolic properties of the nutrients themselves. They have also pointed out that evaluating inter- and intra-macronutrient ratios may provide a measure of dietary macronutrient quality and how proteins, carbohydrates and fats affect health outcomes^(19)^. Improving our knowledge of the relationships between specific macronutrient intake ratios and outcomes may help establish benchmarks for better macronutrient quality and shape future guidelines concerning the prevention and treatment of diseases^(19)^.

In brief, using log-ratio transformations of dietary data seems to be an appropriate approach because it is consistent with the compositional nature of the data themselves, and because the formulation of log-ratios as balances may capture the interdependent dynamics of dietary components. Furthermore, the compositional procedure provides a means of meeting the three important goals of (1) disentangling the relative and absolute aspects of the data; (2) obtaining meaningful estimate of the effects of total energy intake and (3) better controlling confounding factors.
